# The Spinal Instability Neoplastic Score correlates with epidural spinal cord compression -a retrospective cohort of 256 surgically treated patients with spinal metastases

**DOI:** 10.1186/s12891-024-07756-9

**Published:** 2024-08-15

**Authors:** Lukas Bobinski, Joel Axelsson, Jonathan Melhus, Josefin Åkerstedt, Johan Wänman

**Affiliations:** Department of Diagnostics and Intervention, Umeå University, Umeå, Sweden

**Keywords:** Cancer, Spinal instability, Spine metastasis, SINS, ESCC

## Abstract

**Background:**

Bone metastases can compromise the integrity of the spinal canal and cause epidural spinal cord compression (ESCC). The Spinal Instability Neoplastic Score (SINS) was developed in order to evaluate spinal instability due to a neoplastic process. The SINS has reached wide acceptance among clinicans but its prognostic value is still controversial. The aim was to investigate the correlation between the SINS and ESCC and the association between SINS and ambulation before and survival after surgery.

**Methods:**

Correlations were assessed between SINS and grades of ESCC in patients who underwent spine surgery for spinal metastases. CT and MRI were used to calculate SINS and the grades of ESCC respectively. Correlations were analyzed with the Spearman’s correlation test. Postoperative survival was estimated with Kaplan-Meier analysis and survival curves were compared with the log-rank test. The Cox proportional hazard model was used to assess the effect of prognostic variables including age, ambulation before surgery, SINS, and the Karnofsky Performance Status (KPS) as covariates.

**Results:**

The study included 256 patients (196 men and 60 women) with a median age of 70 (24–88) years. The mean SINS was 10. One hundred fifty-two patients (59%) had lost ambulation before surgery. One hundred and one patients had grades 0–2 and 155 patients had grade 3 according to the ESCC-scale. SINS correlated with the grades of ESCC (*p* = 0.001). The SINS score was not associated with ambulation before surgery (*p* = 0.63). The median postoperative survival was 10 months, and there was no difference in postoperative survival between the SINS categories (*p* = 0.25). The ability to walk before surgery and a high KPS were associated with longer postoperative survival.

**Conclusion:**

SINS correlated with grades of ESCC, which implies that higher SINS may be considered as an indicator of risk for developing ESCC. The SINS was not associated with ambulation before or survival after surgery.

## Introduction

The spine is the most common site for skeletal metastasis [1]. The prevalence of spinal metastasis is expected to increase due to an aging population as well as longer survival for patients with a malignant disease [2]. Bone metastases can compromise the integrity of the spinal canal and cause epidural spinal cord compression (ESCC) [3]. Compression of the spinal cord results in clinical symptoms such as pain, para/tetraplegia and incontinence with reduced quality of life as a consequence [4]. If ESCC is diagnosed and treated at its early stage, neurologic deficits can be prevented or even reversed in most patients [5].

Spinal instability, as a result of neoplastic spinal dissemination and destruction, is significantly different from traumatic instability [3]. In 2010, the spine oncology study group published a score known as the Spinal Instability Neoplastic Score (SINS) [6]. SINS consists of the sum of five radiographic parameters (location, bone lesion quality, spinal alignment, vertebral body collapse, and involvement of posterolateral spinal elements) and one clinical parameter (pain) [7]. All the individual scores are summarized and classified into the following categories: stable (0–6 points), potentially unstable (7–12 points), and unstable (13–18 points) [3]. The score has gained wide acceptance among physicians and has demonstrated substantial to excellent intra- and interobserver reliabilities [8, 9]. Despite the fact that SINS facilitated the research about spinal instability due to the neoplastic process, its clinical prognostic value remains controversial [10]. In contrast to most other scoring systems for spinal metastases, SINS estimates a risk of instability and a potential collapse of the vertebral body, thus might be a useful tool for identifying instability in a stage when ESCC can still be prevented [11].

Several studies have evaluated the prognostic value of SINS in relation to survival and neurological outcomes but with conflicting results [12–15]. To our knowledge, no study has yet assessed eventual correlation between SINS and the grades of ESCC in surgically treated patients. Hence, the aim of the present study was to determine the correlation between SINS and ESCC in surgically treated patients with spinal metastases.

## Materials and methods

### Study design and setting

This is a retrospective cohort study that included 256 patients with spinal metastases that underwent surgical treatment between 2003 and 2022 at the Department of Orthopedics, Umeå University Hospital, Sweden.

### Data collection

Data were collected from patients medical records and included age, sex, anatomical level of tumor lesion, type of primary tumor, survival time after surgery, Karnofsky Performance Status scale (KPS) [16], SINS [6], grades of ESCC according to the Epidural Spinal Cord Compression (ESCC)-scale [17], and Frankel score [18] before surgery.

### SINS

Both magnetic resonance imaging (MRI) and computer tomography (CT) were used for the calculation of the SINS as well as for the evaluation of type of compression; bony, epidural tumor or both. In cases with multiple lesions, only the lesion with the highest SINS within operated area was calculated.

### ESCC-scale and neurologic function

The ESCC-scale was used to evaluate the degree of ESCC based on MRI. The Frankel score was used to evaluate the neurological function before surgery, which was further dichotomized as non-ambulatory (Frankel A-C) or ambulatory (Frankel D or E).

### Statistics

Descriptive statistics of continuous variables were expressed as median (range), while categorical data were expressed as numbers and percentages. The chi-square test was used to analyze categorical variables. The postoperative survival was estimated by Kaplan-Meier analysis, and survival curves were compared with the log-rank test. A multivariate cox regression model for survival was used with SINS, age, ambulation before surgery and KPS as covariates. The Spearman’s rank correlation was used to analyze the correlation between SINS and grades of ESCC. A p-value of < 0.05 was considered statistically significant. The statistical analysis was performed using SPSS Statistics software version 25.0.

## Results

The clinical characteristics of the patients are described in Table 1. The study included 256 patients (196 men and 60 women) with a median age of 70 (24–88) years. The most common malignancy was prostate cancer (*n* = 110, 43%) followed by hematological malignancies (*n* = 48, 27%). The thoracic spine was the most common site for the metastatic lesions (*n* = 189) followed by the lumbar spine (*n* = 49). The majority of the patients (*n* = 198) had a KPS $\:\ge\:$70, with 58 patients having a KPS of 50–70.

**Table 1:** Tumor type and clinical characteristics of 256 patients who underwent surgery for pathologic fractures

	Prostate	Hema.	Others	Urogen.	Kidney	Lung	Breast	GI	Sarc.	Thyr.	Mela.	Liver	Unkn.	Total
Number of patients	110	48	6	3	13	17	16	13	7	3	4	4	12	256
Age, median (Min-Max)	73 (50–88)	68 (46–88)	61 (40–74)	66 (56–70)	62 (51–79)	71 (57–79)	72 (40–82)	62 (44–80)	57 (24–72)	71 (66–84)	60 (43–73)	63,5 (61–78)	69 (56–85)	70 (24–88)
Level of compression														
Cervical	2	7	0	1	0	0	2	1	0	1	0	1	1	16
Thoracic	90	35	5	1	8	12	8	11	6	0	4	2	7	189
Lumbar	18	6	1	1	5	3	6	1	1	2	0	1	4	49
Sacral	0	0	0	0	0	2	0	0	0	0	0	0	0	2
Ambulation														0
Yes	25	25	5	2	8	10	7	5	4	1	3	4	5	104
No	85	23	1	1	5	7	9	8	3	2	1	0	7	152
KPS														0
70–100	93	46	6	3	9	9	9	6	4	0	3	3	7	198
50- 70	17	2	0	0	4	8	7	7	3	3	1	1	5	58

Hema, hematological; Urogen, urogenital; GI, gastrointestinal; Sarc, sarcoma; Thyr, thyroid; Mela, melanoma; Unkn, unknown; Others refer to uterus, squamous cell cancer, nasopharynx, mesothelioma, adenocarcinoma; KPS, Karnofsky performance status

### Spinal instability neoplastic score

The mean SINS was 10. Fourteen patients were classified as stable (SINS 0–6), 204 patients classified as potentially unstable (SINS 7–12), and 38 patients classified as unstable (SINS 13–18) (Table 2).

**Table 2:** Spinal instability neoplastic score (SINS)^a^: distribution of patients in the score categories

SINS categories	Score (points)	Number of patients (*n* = 256)
Spine location		
• Junctional (C0-C2, C7-T2, T11-L1, L5-S1)	3	71
• Mobile spine (C3-C6, L2-L4)	2	41
• Semi-rigid (T3-T10)	1	144
• Rigid (S2-S5)	0	0
Mechanical or postural pain		
• Yes	3	197
• No	1	38
• Pain-free lesion	0	21
Bone lesion quality		
• Lytic	2	151
• Mixed lytic/blastic	1	74
• Blastic	0	31
Spinal alignment		
• Subluxation/translation	4	3
• De novo deformity (kyphosis/scoliosis)	2	44
• Normal alignment	0	209
Vertebral body involvement		
• > 50% collapse	3	62
• < 50% collapse	2	47
• No collapse with > 50% of body involved	1	91
• None of the above	0	56
Posterior involvement		
• Bilateral	3	200
• Unilateral	1	48
• None of the above	0	8
Total SINS		14
Stable (0–6 points)		
Potentially unstable (7–12 points)		204
Unstable (13–18 points)		38

^a^ Fisher et al. 2010 [6]

### The correlation between SINS and the ESCC-scale

Out of the 256 patients, 254 (99%) presented with ESCC (ESCC-scale > 0), 101 patients were graded as 0–2, and 155 patients graded as 3 according to the ESCC-scale. The frequency of patients with the highest grade of ESCC (grade 3) was highest in the unstable group (*p* = 0.005). Only 14 out of the 256 patients (5%) met the criteria for stable according to SINS and half of these patients were graded as 3 according to the ESCC-scale (Table 3).

**Table 3:** Grades of metastatic spinal cord compression in relation to the spinal instability neoplastic score (SINS) for 256 patients who underwent spine surgery for spinal metastases

SINS category (points)	ESCC 0–2	ESCC 3	Total
Stable (0–6)	7	7	14
Potentially unstable (7–12)	88	116	204
Unstable (13–18)	6	32	38

ESCC: Epidural spinal cord compression-scale. ESCC grades = Grade 0, bone disease only; Grade 1a, epidural impingent; Grade 1b, deformation of the thecal sac without spinal cord abutment; Grade 1c, deformation with spinal cord abutment, but without cord compression; Grade 2, spinal cord displacement with visible cerebrospinal fluid (CSF); Grade 3, spinal cord compression with no visible CSF. [25]

There was a statistically significant correlation between SINS and grades of ESCC. The Spearman’s correlation coefficient was 0.19 (*p* = 0.002) between SINS and all the different grades of the ESCC-scale, and 0.21 (*p* = 0.001) between SINS and grade 3 of the ESCC.

A bony fragment was part of the compression in 114 patients; most these patients (*n* = 87) had a combination of the compression both from epidural tumor infiltration and a bony component. In 27 patients, the bone component alone comprised the ESCC. There was a statistically significant correlation between higher SINS and the occurrence of a bony fragment as a part of the ESCC with the Spearman’s correlation coefficient of 0.34, *p* < 0.001.

### Neurological function

Before surgery 152 patients (59%) were non-ambulatory (Frankel A-C) and 104 patients (41%) were ambulatory (Frankel D-E). The SINS categories were not associated with ambulation before surgery (*p* = 0.63) (Table 4).

**Table 4:** Ambulation before surgery in relation to the spinal instability neoplastic score (SINS)

SINS category^a^ (points)	Non-ambulatory	Ambulatory	Total
Stable (0–6)	8	6	14
Potentially unstable (7–12)	124	80	204
Unstable (13–18)	20	18	38

Number of patients in each SINS category in relation to ambulatory function before surgery. ^a^ Fisher et al. 2010 [6]

### Survival

The median postoperative survival was 10 months. Patients with hematological malignancies had the longest survival (59 months) followed by breast cancer patients (15 months). The median postoperative survival was 8 months (0–18, 95% CI) for the SINS stable group, 10 months (6–14, 95% CI) for the SINS potentially unstable group, and 14 months (6–22 months, 95% CI) for the SINS unstable group. There was no statistically significant difference in postoperative survival between the SINS groups (*p* = 0.25). In the multivariate cox regression model, ambulation before surgery (*p* = 0.015) and KPS (*p* < 0.001) were associated with a longer survival (Table 5).

**Table 5:** Multivariate Cox regression analysis of covariates in relation to postoperative survival

Group	HR	95% CI	*p*-value
SINS			
Unstable	Ref		
Potentially Unstable	1.3	0.9–1.9	1.3
Stable	1.8	0.9–3.5	0.083
Age	1.0	1.0–1.0	0.26
KPS			
80–100	Ref		
50–70	1.8	1.3–2.4	< 0.001
Ambulation before surgery			
Ambulatory	Ref		
Non-Ambulatory	1.5	1.1–1.9	0.015

*Abbreviations* HR, Hazard Ratio; CI, Confidence Interval; Ref, Reference Category; SINS, Spinal Instability Neoplastic Score; KPS, Karnofsky Performance Status

## Discussion

In this large retrospective study of patients that underwent surgery for spinal metastases, higher SINS values correlated with more severe grades of ESCC.

The progression of metastatic destruction in the spinal column inevitably compromises biomechanical integrity of the vertebrae. The infiltrated vertebra becomes prone to fractures and may collapse and contribute to development of ESCC. Therefore, the correlation between grades of instability and ESCC is relevant in the evaluation of SINS as a tool for assessment of tumor related instability. The evaluation of compression type; bony, epidural tumor or both is also of relevant for the understanding of different compression types and the correlation to SINS. Our results demonstrated a statistically significant correlation between SINS and grades of ESCC as well as between higher SINS and the occurrence of a bony fragment as a part of the ESCC. Furthermore, most patients with the highest grade of ESCC also demonstrated high SINS values. This may imply that the same process of erosion of integrity of spinal column that leads to development of instability may also induce ESCC for most patients. Hence, SINS could be relevant tool for the assessment of tumor-associated instability with an increased degree of ESCC in patients with higher SINS values. Few studies have investigated the association between SINS and ESCC. In a recent prospective cohort study, SINS was identified as the most important factor to predict the onset of symptomatic spinal metastasis, and the authors suggested that patients with SINS > 10 and an estimated survival over 6 months should undergo prophylactic surgery [19]. During the follow-up, 37 patients (29%) experienced symptomatic spinal metastasis, but only 12 of these patients developed ESCC [19]. In a retrospective study that included 299 patients, Lam et al. [20] found that patients with a SINS ≥ 11 after radiotherapy had a more than 2.5-fold risk of spinal adverse events compared to patients with a SINS ≤ 10. During their study period, 98 spinal adverse events occurred, only 11 of these were spinal cord or cauda equina compression [20]. In a retrospective cohort study of 78 patients, Chang et al. [21] did not find any association between SINS and the development of ESCC. Pain and posterolateral involvement, on the other hand, were associated with ESCC that occurred in 27% of patients [21]. The mean SINS of 10 in our study is in line with a multi-institutional retrospective study of 1509 patients that found a mean SINS of 10.7 in operated patients [10].

In general, acute progressing neurological deficit is a strong indication for surgery in patients with spinal metastases. Consequently, the high percentage of ESCC in our study is not surprising. Our findings indicate that a higher SINS score indicates not only spine instability but may also be considered an indicator for risk of development of ESCC. However, the relationship between these conditions is far more complex and requires further investigations.

In the present study, SINS was not associated with ambulation before surgery despite its correlation with grades of ESCC. We can only speculate that other factor such as the cranio-caudal extend of the metastatic lesion, the rate of the expansion of ESCC, as well as the speed of neurological deterioration that were not taken into account in this study might to some extent be an explanation of these findings. Our results are in accordance with the results of Uei et al. [22], who found that the severity of neurological deficit was not correlated to the ESCC-scale in their study of 467 patients.

Several studies have evaluated the association between SINS and postoperative survival with conflicting results [12–15]. The SINS was not associated with postoperative survival in our study, but we found that both ambulation before surgery and KPS were significantly associated with survival, which is in accordance with other studies [23, 24].

The present study has several limitations. The main limitations are linked to the retrospective study design, the lack of a control group, and selection bias since all the patients underwent surgical treatment. The main strength of this study is the large sample size.

## Conclusion

SINS correlated with grades of ESCC, which implies that higher SINS may be considered as an indicator of risk for developing ESCC. The SINS was not associated with ambulation before or survival after surgery.

## Data Availability

The datasets generated during and/or analysed during the current study are available from the corresponding author on reasonable request.
